# Association of hypoadiponectemia with smokeless/dipping tobacco use in young men

**DOI:** 10.1186/s12889-015-2409-7

**Published:** 2015-10-19

**Authors:** Sardar Ahmad, Mohsin Shah, Jawad Ahmed, Aslam Khan, Hamid Hussain, Mary McVey, Asif Ali

**Affiliations:** Department of Physiology, Institute of Basic Medical Sciences, Khyber Medical University, Peshawar, Pakistan; Department of Pharmacology, Institute of Basic Medical Sciences, Khyber Medical University, Peshawar, Pakistan; Institute of Public Health and Social Sciences, Khyber Medical University, Peshawar, Pakistan; School of Life Sciences, College of Medical, Veterinary and Life Sciences, University of Glasgow, Glasgow, Scotland UK; Department of Pathology, Institute of Basic Medical Sciences, Khyber Medical University, Peshawar, Pakistan; Institute of Basic Medical Sciences, Khyber Medical University, Adjacent PDA building, Phase 5, Hayat Abad, Peshawar, Pakistan

**Keywords:** Tobacco, Adiponectin, Nicotine, Dipping tobacco

## Abstract

**Background:**

Low levels of adiponectin, an adipocytokine with anti-diabetic, antiatherogenic and cardioprotective properties, is associated with increased risk of coronary disease in young men. Previous studies have demonstrated that smokeless tobacco is linked with a reduction of plasma adiponectin levels. However, the influence of smokeless tobacco (dipping tobacco) on plasma adiponectin levels still remains unknown. This study was conducted to assess the plasma adiponectin levels in young men who were using dipping tobacco.

**Methods:**

This was a community based study, which consisted of 186 young lean healthy males aged 20 to 35 years. Among these, 96 men were dipping tobacco users (BMI = 23.07 ± 2.68) and 90 were non-dipping tobacco users (BMI = 23.67 ± 1.46). Serum adiponectin levels were assessed by Enzyme Linked ImmunoSorbent Assay (ELISA).

**Results:**

A statistically significant difference in the mean adiponectin level between tobacco dipper and non-dipper groups was observed (*p* = 0.0001). A significant difference between the two groups was also observed in baseline parameters including triglyceride and random blood sugar levels (*p* < 0.05). However, no significant difference was observed between the two groups in other clinical parameters.

**Conclusions:**

Findings of this study suggest that dipping tobacco use was significantly associated with low level of adiponetin in community dwelling young males. This emphasizes the importance of developing community intervention to reduce the use of dipping tobacco, which will reduce the tobacco associated disease burden in the community and will improve public health.

## Background

The adipocyte derived plasma protein adiponectin, also called ARCP30, ADIpoQ, apM1 or GBP28 is a 247 amino acid peptide that accounts for about 0.05 % of total serum proteins [1–3]. The normal concentration of adiponectin in plasma ranges from 5 to 30 μg/ml in males, whereas a higher concentration is found in females [4]. Adiponectin is an important molecule shown to be involved in obesity, metabolic syndrome, cardiovascular disease, lipid metabolism, and hypertension [5–8]. Lower levels of circulating adiponectin have recently been shown to be associated with a number of conditions in humans. Prominent among them are coronary artery disease, myocardial infarction, atherosclerosis, re-stenosis after percutaneous coronary intervention, type 2 diabetes mellitus as well as hypertension [2, 5, 9–13]. Recent studies suggest that adiponectin has anti-atherogenic and anti-inflammatory properties. It is involved in the lowering of many anti-inflammatory cytokines such C-reactive protein, tumor necrosis factor-α and interleukin-6 [14–18].

Tobacco use is a significant contributing risk factor for non-communicable diseases leading to approximately six million deaths worldwide every year [19]. The increasing trend of tobacco use in developing countries is alarming, where 80 % of deaths are expected to occur in next few decades due to toabcco use. [20]. In south Asian countries, the prevalence of tobacco use was estimated to be 72.3, 60.0, 47.3, 34.7, 34.1, 33.6 and 31.6 % in Indonesia, Bangladesh, Maldives, Cambodia, India and Pakistan, respectively [21].

Similar to that of smoking tobacco, smokeless tobacco use was also found equally harmful in a variety of diseases. Kumada et al., reported that coronary artery disease (CAD) was more prevalent in patients with hypoadiponectemia (less than 4 μg/mL) irrespective of other known risk factors like diabetes mellitus, dyslipidemia, hypertension, smoking habit, and BMI in male subjects [10]. Huhtasaari et al., reported smokeless tobacco as a possible risk factor for myocardial infarction in a population based study performed on middle-aged men [22]. Smokeless tobacco was also found to be associated with hypertension in rural Indians [2]. A significant increase in both systolic and diastolic blood pressure was observed in young male population using smokeless tobacco compared to non-users [23]. Wolk et al., reported that nicotine of spit tobacco induced catecholamine release from the adrenal medulla which in turn causes increase in heart rate and blood pressure [24]. Smokeless tobacco (Naswar) use and its association with cancer is well documented. The data from Pakistani hospitals specialized in treating cancer patients showed that 9.9 % of all cancer patients were suffering from oropharyngeal cancer. In addition, with 23.6 % being smokers and 37.4 % being smokeless tobacco users [25].

Smokeless tobacco has not gained the attention of the scientific research community in comparison to smoking, though its use is common all over the world. Majority of dipping tobacco users believe that it is less harmful and that its use can go unnoticed. Dipping tobacco and other forms of smokeless tobacco are widely used in some populations. Grinded tobacco leaves in moist powdered form make dipping tobacco, which is kept between the lower lip and gum and discarded after about 15–30 min. In the local language (Pashto), it is called Naswar (dipping tobacco/moist snuff). It is available locally as loose meshed ground tobacco or as sachets in the developed world.

Peshawar is the capital city of Khyber Pakhtunkhwa, where the vast majority of ethnic Pashtoons reside where 43.79 % of males and 4.4 % of females use dipping tobacco [26]. Several studies have suggested the association of smoking with low levels of adiponectin in plasma, but data regarding dipping tobacco is lacking. Therefore, a case control study was conducted in community dwelling young males to determine if there was any correlation of dipping tobacco usage and levels of plasma adiponectin.

## Methods

### Study design

This was a case control study. The study population consisted of 186 young, lean and apparently healthy male adults. The study participants were divided in to two groups: dipping tobacco users (*n* = 96) and non-dipping tobacco users (*n* = 90). Inclusion criteria were: healthy individuals between 20 and 35 years of age and body mass index (BMI) in the range of 20–25 kg/m^2^. The cases (dipping tobacco users) were those who had history of taking dipping tobacco on a daily basis for a minimum of 5 years regularly with an average of one packet (approximately 50–60 g/day). The controls (non-dippers) were selected from similar matched socio-demographic background and were young healthy subjects with no history of using dipping tobacco or tobacco use by other means. In addition, these control participants were matched to case participants in terms of age and BMI. All the cases and controls selected were young, healthy, lean subjects to remove the possibility of obesity induced hypoadiponectemia or risk of aging associated derangements in physical and biochemical parameters. A structured questionnaire was used in each face to face interview to obtain the relevant information. The study participants were evaluated by medical history for use of alcohol, by physical examination for obesity (BMI >25 kg/m^2^), and routine clinical laboratory investigations to exclude individuals with type 2 diabetes, anemia, liver diseases, chronic renal failure and cardiovascular diseases. Past dipping tobacco users, and subjects with BMI more than 25 were excluded from the study. Study participant on drug(s) which alter hormone levels were also excluded from the study. The prevalence of dipping tobacco among women was very low around 4.4 % and the access to women was difficult due to traditional and cultural restrictions in this area, therefore, women were also excluded from the study [26, 27]. The study was approved by the institution ethical committee of Khyber Medical University, Pakistan. After the nature of the study was explained in detail, informed consent was obtained from all participants.

### Anthropometric parameters

Anthropometric data (weight & height) of the study subjects were collected and BMI was calculated as kg/m^2^. Resting blood pressure was measured using a standard mercury sphygmomanometer. Two blood pressure readings were taken with the participant resting for 10 min, and the mean of the two readings was calculated and used for data analysis.

### Laboratory measurement

Non-fasting venous blood samples (10 ml divided into two 5 ml tubes) were collected in sterile syringes using aseptic technique. The samples were immediately transported to the laboratory (within 2 h) in ice cooled containers for further examination. Serum was separated after centrifugation at 12,000 × *g* and stored at −20 °C for subsequent analysis. Total cholesterol (TC), triglyceride (TG), high-density lipoprotein cholesterol (HDL-C), low-density lipoprotein cholesterol (LDL-C), random blood sugar, serum creratinin were measured in both cases and control groups with a standard protocol using semi-automated chemistry analyzer COBAS C111 (Roche) and kits from the same company.

### Adiponectin assay

Serum low molecular weight adiponectin concentration was determined by ELISA method using the kits supplied by Glory Science Co., Ltd. Research (Del Rio, TX 78840, USA). The kit uses double-antibody sandwich ELISA to assess the level of human adiponectin in serum samples. The wells were pre-coated with human adiponectin monoclonal antibodies. The secondary antibodies consisted of adiponectin antibodies labelled with biotin, and combined with streptavidine-HRP to form an immune complex. After washing completely, 3,3′,5,5′-tetramethylbenzidine (TMB) liquid substrate was added. The TMB substrate changed a blue color when HRP enzyme catalyzed the reaction. The reaction was terminated by the addition of a stop solution and the color change was measured at a wavelength of 450 nm. All the steps of the assay were done according to the manufacturer’s instructions. The results were analyzed by three independent researchers, blinded to the clinico-demographic data of study participants to remove subjective bias. The concentration of adiponectin in the sample was determined by comparing the optical densities of the samples from the standard calibration curve with negative controls. Adiponectin samples were run in duplicate and mean values were calculated for each sample.

### Statistical analysis

Means or proportions of clinical characteristics were analyzed for each group in the study. All data was expressed as means ± standard deviation. Differences between groups were examined for statistical significance using the student’s *t*-test, *p* ≤ 0.05 was considered statistically significant. Unpaired *t*-test (independent sample *t* test) was used to examine the differences in adiponectin levels between the two groups. All analyses were performed using SPSS software, version 19 “SPSS (IBM, Armonk, NY, USA).”

## Results

The study participants were primarily of Pashtoon ancestry, living in the north western areas of Pakistan with the majority using dipping tobacco (about 64 %). The subjects in this study comprised of a total of 186 young, lean, male adults, who were apparently healthy. Among these subjects 96 were dipping tobacco users (28.84 ± 4.77 years old), 90 were non-dipping tobacco users (28.41 ± 4.99 years old), with age range from 20 to 35 years.

Table 1 summarizes the baseline characteristics of each participant. Significant changes in some clinical parameters were observed between cases and control groups while in others, no significant variations were observed. BMI was 23.07 ± 2.68 kg/m^2^ in the dipping tobacco users while it was 23.67 ± 1.46 kg/m^2^ in the non-users. Systolic and diastolic blood pressure in tobacco dippers and non-dippers:119.8 ± 9.67 and 118.61 ± 8.51; 80.36 ± 9.60 and 79.5 ± 8.74 mmHg respectively showing no significant difference. Serum creatinin was 0.78 ± 0.09 in dippers as compared to non-dippers 0.78 ± 0.08 mg/dl (*p* = 0.94). The values of triglycerides were 127.39 ± 33.43 in dippers and 113.81 ± 28.57 mg/dl in non-dippers (*p* = 0.003) showing highly significant difference between the two study groups. Similarly, the values of HDL-C were 48.28 ± 3.74 and 47.22 ± 6.48 mg/dl in dippers and non-dippers respectively (*p* = 0.171). The level of random blood sugar in dippers was 101.8 ± 15.46 and 96.54 ± 10.06 mg/dl in non-dippers (*p* = 0.013). The level of LDL-C was 113.10 ± 18.14 in dippers and 108.70 ± 23.11 mg/dl in non-dippers (*p* = 0.149) showing statistically no significant difference.

**Table 1:** Baseline characteristics of young adults in dipping tobacco users and nondippers

Characteristics	Dippers (*n* = 96)	Nondippers (*n* = 90)	*p* value
Age (years) mean ± SD	28.84 ± 4.77	28.41 ± 4.99	0.547
BMI, mean ± SD, kg/m^2^	23.07 ± 2.68	23.67 ± 1.46	0.060
Systolic blood pressure, mean ± SD, mmHg	119.8 ± 9.67	118.61 ± 8.51	0.379
Diastolic blood pressure, mean ± SD, mmHg	80.36 ± 9.60	79.5 ± 8.74	0.523
Random blood sugar, mean ± SD, mg/dl	101.79 ± 15.46	96.54 ± 10.06	**0.013**
Serum creatinine, mean ± SD, mg/dl	0.78 ± 0.09	0.78 ± 0.08	0.941
Total cholesterol, mean ± SD, mg/dl	161.38 ± 19.53	161.16 ± 25.22	0.947
TG, mean ± SD, mg/dl	127.39 ± 33.43	113.81 ± 28.57	**0.003**
HDL cholesterol, mean ± SD, mg/dl	48.28 ± 3.74	47.22 ± 6.48	0.171
LDL cholesterol, mean ± SD, mg/dl	113.10 ± 18.14	108.70 ± 23.11	0.149
Serum adiponectin, mean ± SD, μg/ml	3.23 ± 2.07	12.94 ± 10.50	**0.0001**

*Abbreviations*: *BMI* Body Mass Index, *TG* Triglycerides, *HDL* High Density Lipoproteins, *LDL* Low Density Lipoproteins. For these variables, data are presented as mean ± SD and student’s *t*- test was applied. The letters in bold shows the significant difference between the two study groups

**Fig. 1:**
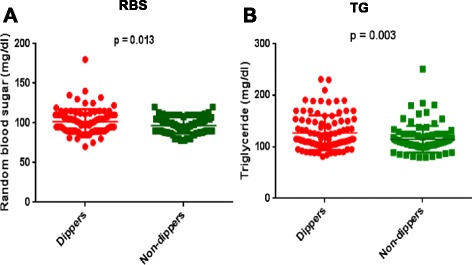
Scatter plots of serum random blood sugar (**a**) and triglyceride (**b**) (individual’s data for each subject) in smokeless tobacco dippers (*n* = 96) and non-dippers (*n* = 90). The *horizontal lines* represent the means between the tobacco dippers and non-dippers. *P* values represent the significance of the effect of random blood sugar (**a**) triglyceride (**b**) levels between the two study groups

Figure 1 shows the scatter plots of the difference in the level of random blood sugar and triglycerides between the dipping tobacco users and non-dippers. It is clear that the level of random blood sugar significantly changed (*p* = 0.013) in dippers as compared to non-dippers (Fig. 1a). Similarly, the values of triglycerides were also significantly changed (*p* = 0.003) in both groups (Fig. 1b).

**Fig. 2:**
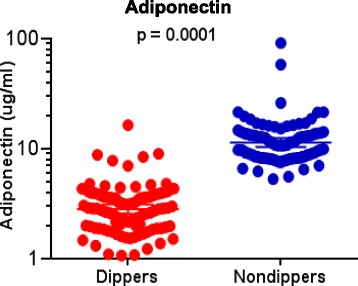
Serum adiponectin concentrations in dipping tobacco users and non-users. Individual’s data for each subject in smokeless tobacco dippers (*n* = 96) and non-dippers (*n* = 90). The *horizontal lines* represent the means between the tobacco dippers and non-dippers. *P* values represent the significance of the effect of adiponectin concentration between dipping tobacco users and non-users

Figure 2 shows adiponectin levels in dippers and non-dippers. The most promising finding in our study was the low level of adiponectin in dippers compared to those of non-dippers. The mean serum adiponectin level was 3.23 ± 2.07 in dippers, while it was 12.94 ± 10.50 in non-dippers (*p* = 0.0001, independent sample *t* test). Thus a statistically significant lower level of serum adiponectin was observed in dippers compared to non-dippers. No association of adiponectin was observed with other clinical parameters, e.g. hypertension, HDL-C and raised triglyceride in both tobacco dippers and non-dippers.

## Discussion

This study was designed to assess the effect of dipping tobacco use on serum adiponectin concentration. Our results showed a significantly lower serum adiponectin level in healthy young males who were using dipping tobacco for a minimum of 5 years regularly with an average of one packet (approximately 50–60 g) daily. In contrast to the published literature we did not find any association of lower adiponectin with hypertension (both systolic and diastolic), serum creatinin, total cholesterol, LDL-C and HDL-C levels [27, 28]. The discrepancies in these parameters might probably be due to the inclusion of young lean adults in the current study. The present study is therefore unique because we considered lean young individuals who were in the highly productive and highly resistant ages of their lives compared to previous studies which included subjects with age above 40 years [27–29]. The clinical parameters including blood pressure, serum creatinin, total cholesterol, LDL-C and HDL-C were not significantly different in dipping tobacco users relative to those of age matched non-dipping control group (Table 1). The level of adiponectin is reported to increase in nephritic and renal failure [30, 31]. Therefore, we measured serum creatinin in order to exclude any renal dysfunction in our study groups. Astonishingly, we did not detect any significant change in the levels of HDL-C, LDL-C, serum creatinine and total cholesterol in the present study.

The levels of triglyceride were significantly different between tobacco dippers and non-dippers (Table 1 shown in bold face letter). Our results support the previous literature where adiponectin was found to be inversely associated with triglyceride levels [32, 33].

It has been reported previously that cigarette smoking is associated with low plasma adiponectin levels [9, 34], however, to the best of our knowledge, no study has explored the relationship between smokeless tobacco use and serum adiponectin level in young adult males. Therefore, we hypothesized that similar to smoking tobacco; smokeless tobacco might be associated with low level of adiponectin in community-dwelling young adults. Our results support the previous literature on tobacco induced hypo-adiponectemia. Kawamoto et al., reported significantly lower adiponectin level in community dwelling Japanese men aged 20 to 89 years using smoking tobacco [27]. An interesting study showed a significant difference in adiponectin level in current smokers, those who have stopped smoking and those who had never smoked [9].

Importantly, some studies have shown an increase in plasma adiponectin level following cessation of smoking [35, 36]. In a study by Otsuka et al., cessation of smoking was associated with increased plasma adiponectin level in 47 non smokers and 25 current smokers with a 6 month follow-up period [35]. Barrios et al. reported that low level of adiponectin (≤4.5 μg/mL) is associated with adverse cardiovascular health [37], though the mean age and BMI of our study participants was less than than the participants of that study.

These findings have led us to explore new possibilities of the association between dipping tobacco and plasma adiponectin levels. Adiponectin is a major contributor in insulin sensitivity and in decreasing the risk of diabetes and coronary heart diseases. Dipping tobacco is commonly used in ethnic Pashtoon areas in Pakistan and Afghanistan. Our study showed an inverse relationship of dipping tobacco with adiponectin level. Clearly, this association would lead to adverse cardiovascular health. Thus this novel finding could help the general public and health authorities understand the association of dipping tobacco with adverse cardiovascular health. In addition, this study will also help designing future studies on exploring the underlying molecular mechanisms responsible for hypoadiponectemia.

Our study has certain limitations. In this study we were unable to compare our results with individuals using smoking tobacco. The inclusion of smoking tobacco group would have made the results of this study more informative. This would have provided us with evidence on adiponectin level in three groups: dipping tobacco users; smoking tobacco users; and both dipping and smoking tobacco users. However, due to limited funding we were not able to accomplish this part of the study but we plan to explore this aspect in a future study. In addition, the smokeless tobacco was not biochemically verified. Adiponectin may lower C-reactive proteins as well as a variety of other inflammatory biomarkers as previously reported [14–18]. However, we were not able to measure the levels of these inflammatory biomarkers in our study. Another limitation of our study was the non-fasting venous blood sampling due to restricted access to individuals and to convince them for providing fasting blood samples. We were also unable to assess the dose dependent response of dipping tobacco in changing the biochemical parameters. Thus to explore the aforementioned associations future studies are recommended.

## Conclusions

This study showed that the serum adiponectin levels were significantly lower in the dipping tobacco users as compared to non-users in adult lean males. These findings support a critical association of hypoadiponectemia with dipping tobacco use in community-dwelling youth. Consequently, the results of this study provide evidence that will increase the awareness of the general public regarding the adverse effects associated with dipping tobacco use. This will also hopefully help the public health authorities in deciding what measures to take to reduce the use of dipping tobacco and reduce the associated health burden. Moreover, as the level of adiponectin is significantly lowered in dipping tobacco users than non-dippers, it might aid in the early diagnosis of clinical situations (e.g. cardiovascular health).
